# Enhancing patient-centered information on implant dentistry through prompt engineering: a comparison of four large language models

**DOI:** 10.3389/froh.2025.1566221

**Published:** 2025-04-07

**Authors:** John Rong Hao Tay, Dian Yi Chow, Yi Rong Ivan Lim, Ethan Ng

**Affiliations:** ^1^ Department of Restorative Dentistry, National Dental Centre Singapore, Singapore, Singapore; ^2^ Health Services and Systems Research Programme, Duke-NUS Medical School, Singapore, Singapore; ^3^ Private Practice, Royce Dental Group, Singapore, Singapore; ^4^ Centre for Oral Clinical Research, Barts and the London School of Medicine and Dentistry, Queen Mary University of London, London, United Kingdom

**Keywords:** large language models, GPT, artificial intelligence, dental implants, peri-implantitis, prompt engineering, dental

## Abstract

**Background:**

Patients frequently seek dental information online, and generative pre-trained transformers (GPTs) may be a valuable resource. However, the quality of responses based on varying prompt designs has not been evaluated. As dental implant treatment is widely performed, this study aimed to investigate the influence of prompt design on GPT performance in answering commonly asked questions related to dental implants.

**Materials and methods:**

Thirty commonly asked questions about implant dentistry – covering patient selection, associated risks, peri-implant disease symptoms, treatment for missing teeth, prevention, and prognosis – were posed to four different GPT models with different prompt designs. Responses were recorded and independently appraised by two periodontists across six quality domains.

**Results:**

All models performed well, with responses classified as good quality. The contextualized model performed worse on treatment-related questions (21.5 ± 3.4, *p* < 0.05), but outperformed the input-output, zero-shot chain of thought, and instruction-tuned models in citing appropriate sources in its responses (4.1 ± 1.0, *p* < 0.001). However, responses had less clarity and relevance compared to the other models.

**Conclusion:**

GPTs can provide accurate, complete, and useful information for questions related to dental implants. While prompt designs can enhance response quality, further refinement is necessary to optimize its performance.

## 1 Introduction

Dental implants usage has increased dramatically over the last two decades (1). In the United States, the proportion of individuals with at least one dental implant rose from 0.7% in 1999–2000 to 5.7% in 2015–2016, with an annual increase of 14% (1). The largest absolute increase occurred among those aged 65–74 at 12.4%, and projections suggest that dental implant prevalence in the United States could reach as high as 23% by 2026 (1). However, despite advances in surgical technique and prosthetic capabilities, cumulative factors in susceptible individuals can lead to peri-implant disease (2). Peri-implant disease is prevalent, with peri-implantitis affecting approximately 19.5% of patients and 12.5% of implants, though estimates vary based on clinical case definitions (3). Some studies have reported even higher prevalence rates, with peri-implantitis affecting up to 56.6% of patients and 27.9% of implants (4–6). It has also been found that patients often have unrealistic high expectations of dental implant therapy (7–9), and have a low awareness of maintenance strategies and dental implant-related complications (10, 11). This may be partly attributed to patients relying on non-credible information sources (12).

Large language models (LLMs) may potentially be used as an educational tool for patients. LLMs represent a significant advancement in artificial intelligence (AI), particularly in the area of natural language processing. Built on deep neural networks, LLMs can generate human-like text, due to its training on vast amounts of massive text databases. Many modern LLMs, such as OpenAI's ChatGPT, Google's Gemini, and Meta's Llama, possess “few-shot” and “zero-shot” learning capabilities, enabling them to generate human-like text with minimal or even no fine-tuning (13, 14). This is achieved through self-supervised learning, where models learn patterns in language to predict text based on its surrounding context. In healthcare, LLMs have gained considerable attention due to its potential in assisting in diagnosis, treatment planning, and providing medical advice (15–17). Large language models have demonstrated a performance level approximate to a passing grade in dental exams (18, 19), with some models being capable of outperforming dental residents (20). This may have utility in clinical care by assisting dental providers in giving advice to patients. Self-diagnosis rates are highly prevalent, with over one-third of individuals utilizing the internet for health information (21). Given this trend, it is likely that patients will use internet chatbots to answer dental-related queries (22, 23).

Although there have been significant advances in LLMs, their performance can still be improved (14, 24). Prompt engineering is a new field which aims to generate more accurate and consistent responses by creating prompts to guide the model's reasoning process. It is a way of designing instructions to guide a language model's reasoning, giving more accurate responses. For example, prompting methods such as encouraging the model to break down complex problems into intermediate reasoning steps, to “think step-by-step” (chain of thought prompting), or generating multiple responses to the same prompt and selecting the most consistent answer (self-consistency prompting), can enhance LLM performance. However, its effectiveness can still vary widely depending on the prompt design (24, 25). This underscores the need for tailoring prompting strategies to achieve optimal outcomes. To the authors' best knowledge, no studies within the field of Dentistry have compared different prompting strategies in assessing the performance of a Generative Pre-trained Transformer (GPT). A GPT is a type of LLM designed to produce content by comprehending text within a conversation. This capability may be leveraged to provide dental education for patients. As dental implant therapy is a commonly performed procedure in clinical practice, the aim of this study was to investigate the influence of prompt design on GPT performance, using frequently asked questions about dental implants as a test example.

## 2 Materials and methods

One of the state-of-the-art LLMs is the GPT-4o model (14). The programming environment utilized Python 3.10, using the Anaconda 3 distribution, an open-source platform. Interaction with the GPT model was managed via the OpenAI Application Programming Interface (API), enabling controlled input delivery and output retrieval from the GPT model. Four methods of prompt engineering were used: input-output prompting, zero-shot-chain of thought prompting, zero-shot chain of thought prompting with instruction-tuning, and a contextualized model augmented with a dental knowledge base. Input-output prompting is a method of prompt engineering that defines the input and output that the GPT is to generate (25). Zero-shot-chain of thought prompting encourages the model to think “step-by-step” in its reasoning process (26). Instruction-tuning instructs the model to follow specific instructions, and in addition temperature control was set to 0 to achieve the least stochastic (i.e., random) responses (27). A contextualized model in this instance involves processing domain-specific clinical practice guidelines into a knowledge base. The guidelines identified for this study comprised of the latest S3-level clinical practice guidelines for the treatment of Stage I-III periodontitis (28); Stage IV periodontitis (29); and peri-implant diseases (30). These documents were uploaded into the OpenAI API and made accessible for retrieval. A Retrieval-Augmented Generation approach was implemented to dynamically extract relevant content from the knowledge base during interactions. This ensured that responses were based on the S3-level recommendations, rather than relying solely on the model's pre-trained knowledge. The GPT model was asked to assume the role of a general dentist, and explicit instructions were given to each of the models. Full details of the prompts are detailed in Supplementary Table S1.

Three dental specialist fellows (J.R.H.T., E.N., Y.R.I.L.) and one resident (D.Y.C.) in periodontology collaborated closely and compiled a list of 30 questions related to dental implant therapy. The number of questions was selected in line with the exploratory nature of this study, aimed at identifying core issues in implant dentistry (31, 32). This was initially derived from the frequently asked questions section of reputable online sources of dental-related information, namely the European Federation of Periodontology, American Academy of Periodontology, British Society of Periodontology and Implant Dentistry, Singapore Health Services, Academy of Australian and New Zealand Prosthodontists, and Australian and New Zealand Academy of Periodontists (33–38). The initial set of questions were then refined by all members of the study team based on their shared experience in encountering commonly encountered patient enquiries on dental implants, and categorized into question domains related to patient selection, associated risks, peri-implant disease symptoms, dental implant treatment for missing teeth, prevention, and prognosis (Table 1).

**Table 1:** List of questions posed to GPT models.

Question Number	Section 1: Patient selection
1	Who is an ideal candidate for dental implants?
2	Who should not receive dental implants?
3	Can I still have dental implants if I am a smoker?
4	Does having high cholesterol or hypertension affect my eligibility to have implants done?
5	If I am on anti-resorptive medication for osteoporosis, does this mean I cannot have dental implants done?
6	Am I suitable for dental implants if I am a diabetic?
7	Can I still have dental implants if I have previously received head and neck radiation?
	Section 2: Associated risks
8	What are the risks of dental implant surgery?
9	What is peri-implant disease?
10	Who is at risk of peri-implant disease?
11	Can dental implants fail?
	Section 3: Symptoms
12	What are the possible complications of dental implant therapy and how do I spot them?
13	What are the symptoms of peri-implant mucositis?
14	What are the symptoms of peri-implantitis?
	Section 4: Treatment
15	Can you describe the process of dental implant surgery?
16	What additional procedures may be needed for less straightforward dental implant cases?
17	When would bone grafting procedures in conjunction with dental implant therapy be recommended?
18	What are all the stages of dental implant treatment and how long does it take to complete a standard case?
19	Please specify the average treatment time in more complex cases where a staged approach with bone grafting is required?
20	How soon can my implant be restored with a crown?
21	Do I qualify for immediate implants?
22	What are the alternatives to dental implants?
23	What is the treatment for peri-implant diseases?
24	When should my implant be removed?
	Section 5: Prevention
25	Can peri-implant disease be prevented?
26	How are dental implants professionally maintained?
27	How should I care for my implant?
	Section 6: Prognosis
28	How long do dental implants last for?
29	What is considered successful dental implant therapy?
30	What is the success rate following treatment of peri-implant diseases?

The responses for each of the 30 questions were extracted into a standardized form across all four models. To account for run-to-run variation, each query was presented three times to each model. The identities of the models were masked from the raters (E.N. and Y.R.I.L), who assessed each model over four different days with a 72-h wash-out period between evaluations to minimize bias and carryover effects. The Quality Analysis of Medical AI (QAMAI) tool, a validated tool developed to evaluate the quality of health information provided by AI within the context of dentistry and otorhinolaryngology, was utilized (39). The raters each had a minimum of eight years in the practice of periodontology, and independently assessed each response. Responses were evaluated against six quality criteria, namely: accuracy, clarity, relevance, completeness, provision of sources of references, and usefulness, using a scale from 1 to 5: 1 (“strongly disagree”), 2 (“disagree”), 3 (“neutral”), 4 (“agree”), and 5 (“strongly agree”).

### 2.1 Statistical analysis

Average scores and standard deviations were calculated for each of the four models, with further subgroup analysis according to the question and quality domains. To assess for significant differences in scores between models, the Kruskal–Wallis rank sum test was used for overall and domain-specific scores. If significant differences were detected in specific question or quality domains, Dunn's *post hoc* multiple comparison test was conducted. Proportions of response categories (i.e., strongly agree, agree, neutral, disagree, strongly disagree) were compared using a two-tailed Pearson's chi-squared test. Scores from each model were categorized into “pass” and “fail” responses. Ratings of 'strongly disagree', “disagree”, and “neutral” were classified as a “fail”, while ratings of “agree” and 'strongly agree' were classified as a “pass”. Proportions of pass and fail responses were calculated for each model across question domains and quality domains. Fisher's exact test was conducted to identify significant associations between response status and model type. *Post hoc* pairwise tests were conducted between model pairs for domains with significant results. Sensitivity analysis was conducted by recalculating total scores by taking the lower score from the two raters. A *p*-value of <0.05 was considered statistically significant, with adjustments for Bonferroni correction where needed. Statistical analysis was done using R (version 4.3.2, R Core Team, Vienna, Austria).

As synthetic data was utilized, ethical approval was not required under the local Human Biomedical Research Act regulations (40). The study was conducted in accordance with the 2024 revision of the Declaration of Helsinki.

## 3 Results

**Figure 1:**
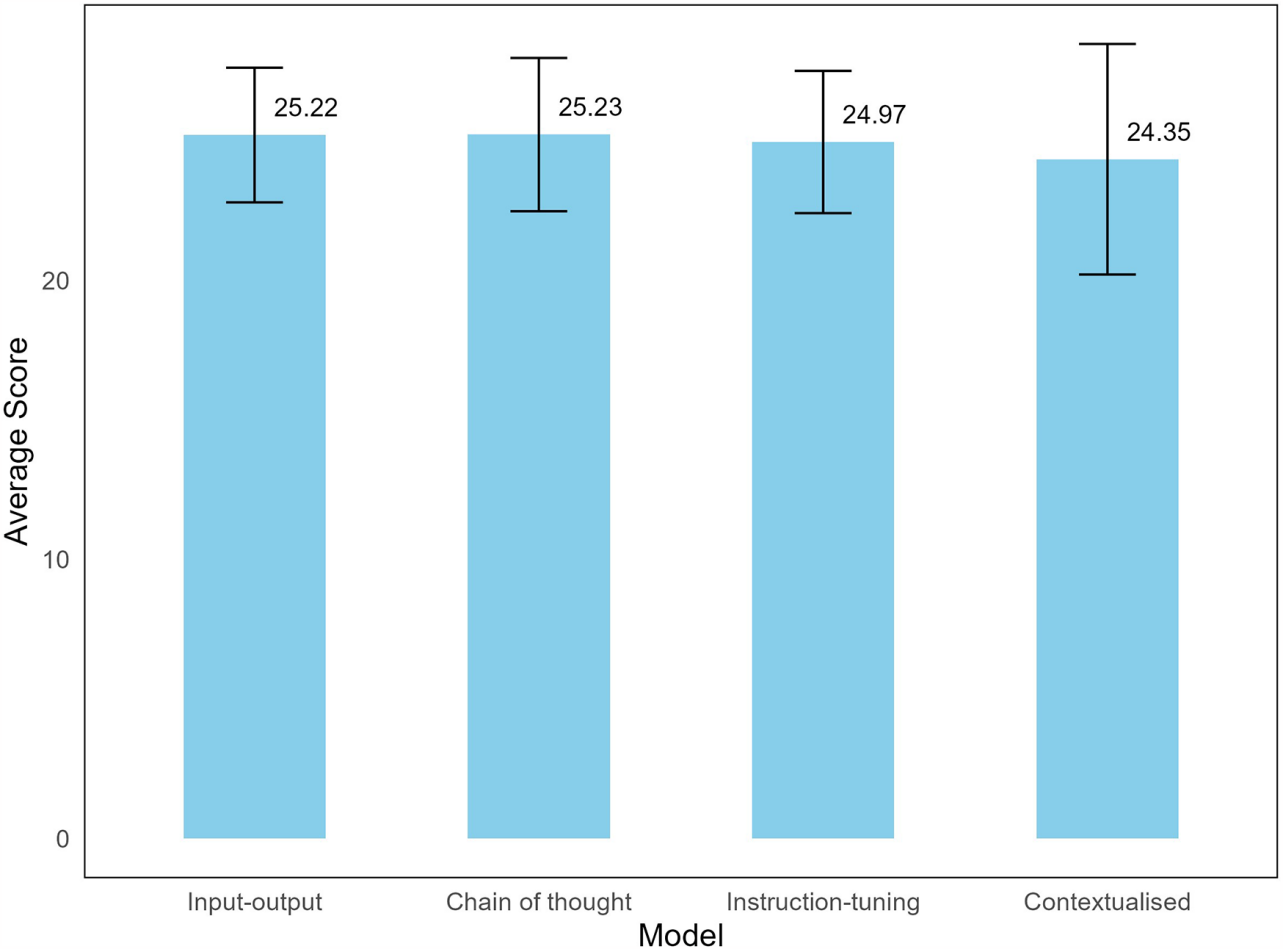
Average scores of LLM models to dental implant-related questions. A maximum of 30 points can be scored for each question.

Using a two-way consistency model, the intraclass correlation coefficient between the two raters indicated good agreement at 0.73 (95% CI: 0.69–0.76). The average scores for all models were relatively high, with most responses across question domains rated as 4 (“agree”) or 5 (“strongly agree”), indicating all models were of very good quality overall according to the QAMAI tool (Figure 1). Run-to-run variations were minimal, showing no difference in scores. The Kruskal–Wallis rank sum test showed no significant differences in average scores across the four models (*p* = 0.933) (Figure 1). Pearson's Chi-squared test did not reveal a statistically significant difference (*p* = 0.10) in response to distributions (i.e., strongly agree, agree, neutral, disagree, strongly disagree) across models. Separate Chi-squared tests for each response category indicated a significant difference in the “disagree” category across models, and although pairwise comparisons showed that the contextualized model received significantly more “disagree” responses compared to input-output model (*p* = 0.016), this result was not significant at the Bonferroni-adjusted level (adjusted *α* = 0.01).

**Figure 2:**
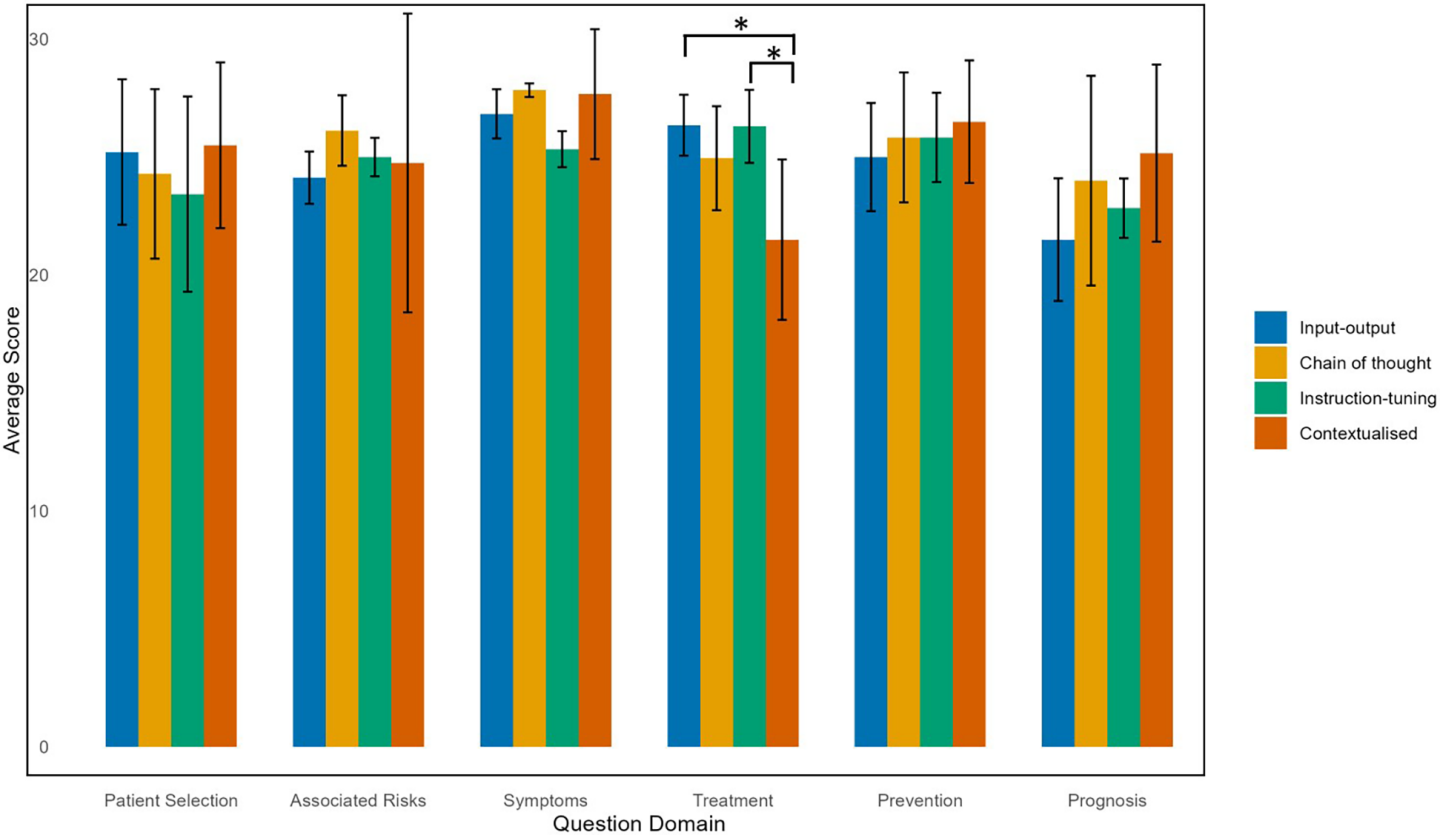
Average scores of LLM models according to question domain.

When categorized according to question domain, the contextualized model had a lower average score of 21.5 ± 3.4 in the Treatment domain, with almost 12% of quality criteria rated as either 1 ('strongly disagree’) or 2 (“disagree”) (Table 2). A statistically significant difference in scores within the Treatment domain was confirmed by the Kruskal–Wallis test, and Dunn's *post hoc* test, indicated that the contextualized model had statistically significantly lower total scores in the Treatment domain compared to input-output model (26.4 ± 1.3, *p* = 0.0036) and chain of thought with instruction-tuning model (25.0 ± 2.3, *p* = 0.0051) after Bonferroni correction (Figure 2). In the Prognosis domain, the input-output model performed the worst with an average score of 21.5 ± 2.6, with nearly 35% of quality criteria rated with a score of 1–3 (“strongly disagree”, “disagree”, “neutral”). However, this difference was not statistically significant (Figure 2, Supplementary Table S2).

**Table 2:** Distribution of response scores by question domains across models.

Domain	Number of questions	Input-output model, *n* (%)				Chain of thought model, *n* (%)					
		Strongly disagree	Disagree	Neutral	Agree	Strongly agree	Strongly disagree	Disagree	Neutral	Agree	Strongly agree
Patient selection	7	2 (2.4)	0 (0.0)	15 (17.9)	29 (34.5)	38 (45.2)	2 (2.4)	2 (2.4)	16 (19.1)	34 (40.5)	30 (35.7)
Associated risks	4	3 (6.3)	1 (2.1)	5 (10.4)	22 (45.8)	17 (35.4)	0 (0.0)	0 (0.0)	4 (8.3)	23 (47.9)	21 (43.8)
Symptoms	3	0 (0.0)	0 (0.0)	5 (13.9)	9 (25.0)	22 (61.1)	0 (0.0)	0 (0.0)	6 (16.7)	1 (2.8)	29 (80.6)
Treatment	10	0 (0.0)	1 (0.8)	13 (10.8)	44 (36.7)	62 (51.7)	5 (4.2)	4 (3.3)	13 (10.8)	43 (35.8)	55 (45.8)
Prevention	3	2 (5.6)	0 (0.0)	1 (2.8)	20 (55.6)	13 (36.1)	0 (0.0)	0 (0.0)	2 (5.6)	21 (58.3)	13 (36.1)
Prognosis	3	6 (16.7)	0 (0.0)	6 (16.7)	15 (41.7)	9 (25.0)	2 (5.6)	2 (5.6)	3 (8.3)	16 (44.4)	13 (36.1)
Domain	Number of questions	Instruction-tuning model, *n* (%)				Contextualized model, *n* (%)					
		Strongly disagree	Disagree	Neutral	Agree	Strongly agree	Strongly disagree	Disagree	Neutral	Agree	Strongly agree
Patient selection	7	1 (1.2)	5 (6.0)	13 (15.5)	43 (51.2)	21 (25.0)	0 (0.0)	3 (3.6)	12 (14.3)	30 (35.7)	39 (46.4)
Associated risks	4	0 (0.0)	0 (0.0)	6 (12.5)	28 (58.3)	14 (29.2)	3 (6.3)	5 (10.4)	1 (2.1)	13 (27.1)	26 (54.2)
Symptoms	3	0 (0.0)	0 (0.0)	7 (19.4)	14 (38.9)	15 (41.7)	0 (0.0)	0 (0.0)	0 (0.0)	14 (38.9)	22 (61.1)
Treatment	10	2 (1.7)	4 (3.3)	7 (5.8)	40 (33.3)	67 (55.8)	3 (2.5)	12 (10.0)	35 (29.2)	52 (43.3)	18 (15.0)
Prevention	3	0 (0.0)	0 (0.0)	4 (11.1)	17 (47.2)	15 (41.7)	0 (0.0)	0 (0.0)	1 (2.8)	19 (52.8)	16 (44.4)
Prognosis	3	6 (16.7)	0 (0.0)	0 (0.0)	19 (52.8)	11 (30.6)	1 (2.8)	0 (0.0)	4 (11.1)	17 (47.2)	14 (38.9)

Each question within a question domain is evaluated across six quality criteria by the two raters. For instance, with four questions in a domain, the total number of assessments would equal 48.

**Figure 3:**
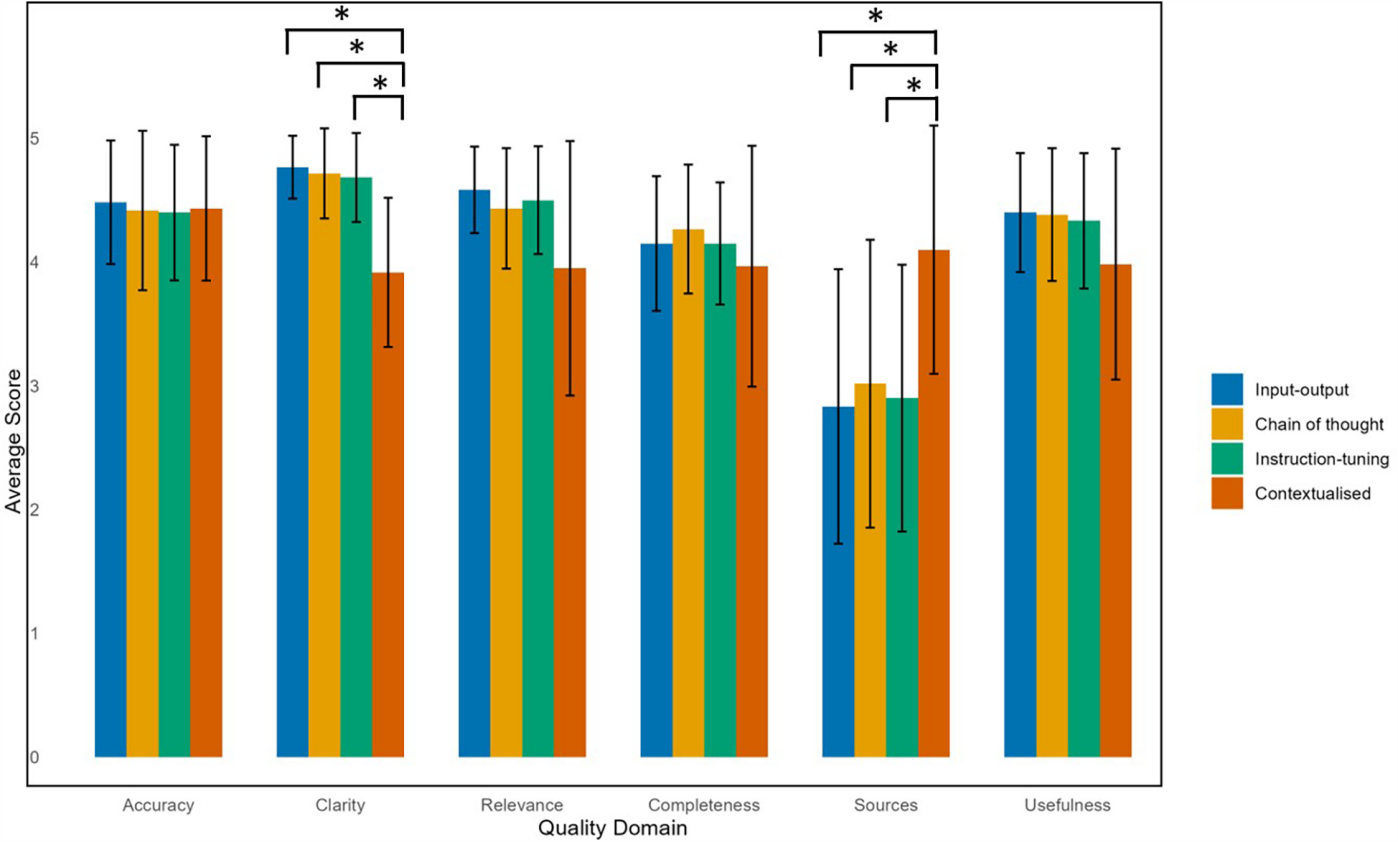
Average scores of LLM models according to QAMAI quality domain.

Comparing across quality domains, the input-output, zero-shot chain of thought, and instruction-tuned models performed poorly in citing appropriate sources in its responses, with around a quarter of responses being scored with a 1 (“strongly disagree”) or 2 (“disagree”) (Table 3). In contrast, the contextualized model scored better in source citation, with 78% of questions being rated with a 4 (“agree”) or 5 (“strongly agree”). The Kruskal–Wallis test confirmed a statistically significant difference, with the contextualized model scoring significantly higher than all other models in source citation and referencing (4.1 ± 1.0, *p* < 0.001) (Figure 3).

**Table 3:** Distribution of response scores across quality domains for each model by both raters.

Domain	Input-output model, *n* (%)					Chain of thought model, *n* (%)				
	Strongly disagree	Disagree	Neutral	Agree	Strongly agree	Strongly disagree	Disagree	Neutral	Agree	Strongly AGREE
Accuracy	0 (0.0)	0 (0.0)	4 (6.7)	23 (38.3)	33 (55.0)	0 (0.0)	1 (1.7)	5 (8.3)	22 (36.7)	32 (53.3)
Clarity	0 (0.0)	0 (0.0)	0 (0.0)	14 (23.3)	46 (76.7)	0 (0.0)	0 (0.0)	1 (1.7)	15 (25.0)	44 (73.3)
Completeness	0 (0.0)	0 (0.0)	8 (13.3)	35 (58.3)	17 (28.3)	0 (0.0)	0 (0.0)	6 (10.0)	32 (53.3)	22 (36.7)
Relevance	0 (0.0)	0 (0.0)	0 (0.0)	25 (41.7)	35 (58.3)	0 (0.0)	0 (0.0)	3 (5.0)	28 (46.7)	29 (48.3)
Sources	13 (21.7)	2 (3.3)	30 (50.0)	12 (20.0)	3 (5.0)	9 (15.0)	7 (11.7)	25 (41.7)	12 (20.0)	7 (11.7)
Usefulness	0 (0.0)	0 (0.0)	3 (5.0)	30 (50.0)	27 (45.0)	0 (0.0)	0 (0)	4 (6.7)	29 (48.3)	27 (45.0)
Domain	Instruction-tuning model, *n* (%)					Contextualized model, *n* (%)				
	Strongly disagree	Disagree	Neutral	Agree	Strongly agree	Strongly disagree	Disagree	Neutral	Agree	Strongly agree
Accuracy	0 (0.0)	1 (1.7)	2 (3.3)	29 (48.3)	28 (46.7)	0 (0.0)	0 (0.0)	6 (10.0)	22 (36.7)	32 (53.3)
Clarity	0 (0.0)	0 (0.0)	1 (1.7)	17 (28.3)	42 (70.0)	1 (1.7)	1 (1.7)	10 (16.7)	38 (63.3)	10 (16.7)
Completeness	0 (0.0)	1 (1.7)	3 (5.0)	42 (70.0)	14 (23.3)	1 (1.7)	6 (10.0)	9 (15.0)	22 (36.7)	22 (36.7)
Relevance	0 (0.0)	0 (0.0)	2 (3.3)	26 (43.3)	32 (53.3)	2 (3.3)	5 (8.3)	11 (18.3)	18 (30.0)	24 (40.0)
Sources	9 (15.0)	6 (10.0)	27 (45.0)	14 (23.3)	3 (5.0)	2 (3.3)	3 (5.0)	8 (13.3)	21 (35.0)	26 (43.3)
Usefulness	0 (0.0)	1 (1.7)	2 (3.3)	33 (55.0)	24 (40.0)	1 (1.7)	5 (8.3)	9 (15.0)	24 (40.0)	21 (35.0)

Although the contextualized model had more ratings of 1–3 ('strongly disagree’, “disagree”, “neutral”) in the clarity, relevance, and usefulness domains compared to the other models, *post hoc* testing revealed it scored significantly lower in clarity (3.9 ± 0.6) compared to the input-output (4.8 ± 0.3), chain of thought (4.7 ± 0.4), and instruction-tuned models (4.7 ± 0.4) (Supplementary Table S2).

**Figure 4:**
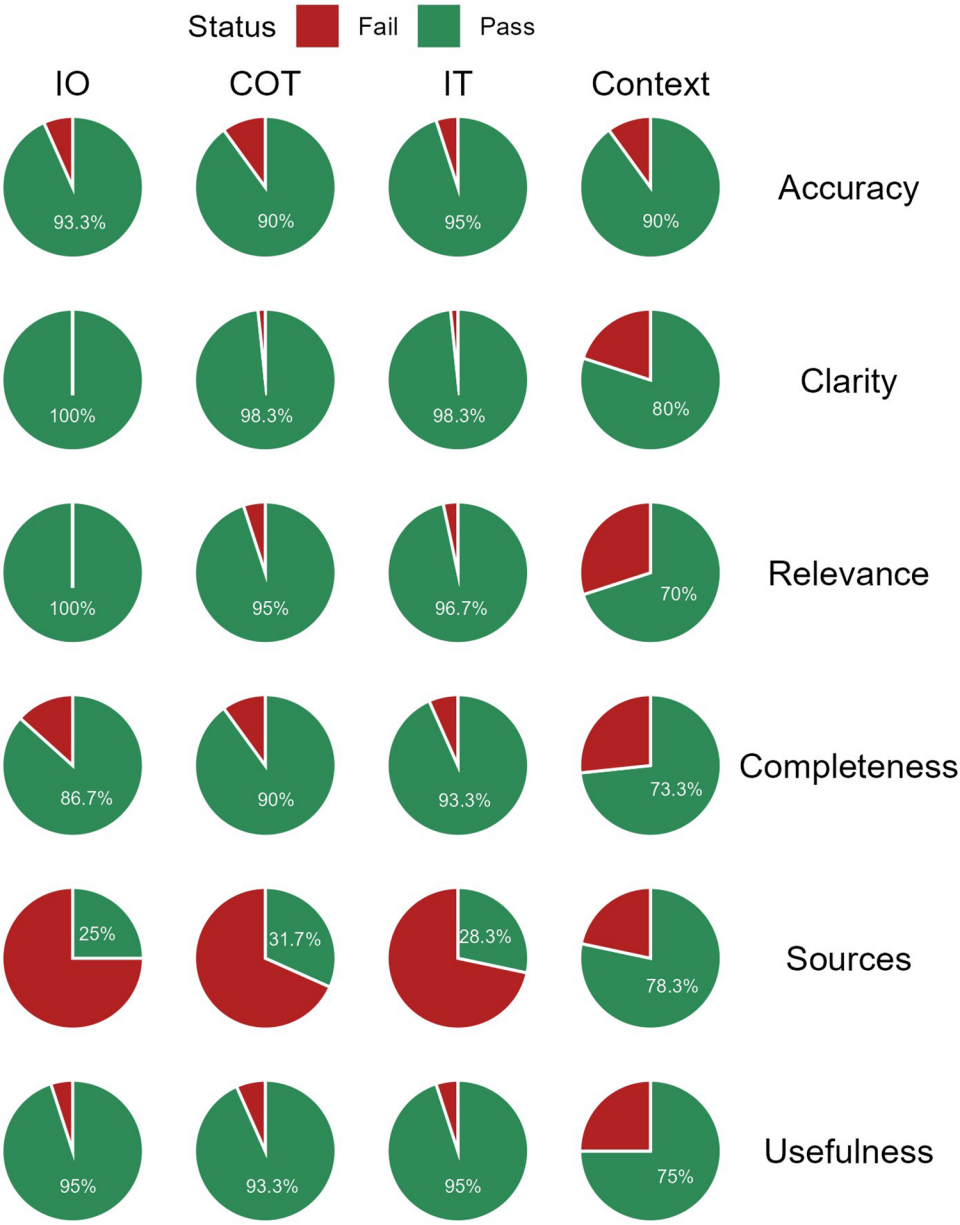
Proportion of “Pass” and “Fail” responses by quality domain and model. IO, input-output; COT, chain of thought; IT, instruction-tuning; Context, contextualized.

When responses were dichotomized into pass and fail categories, the contextualized model had significantly lower pass rates in the Treatment domain [58.3% (95% CI: 49.4–66.8%)] compared to the other models. The contextualized model displayed significantly higher pass rates to the other models when citing relevant sources [78.3% (95% CI: 66.4–86.9%)]. However, it showed lower pass rates in clarity and relevance to the other models (Figure 4, Supplementary Table S3).

Sensitivity analysis was conducted using the lower of the two scores between raters to re-calculate the total scores for each model. The overall distribution of responses was similar across models, with no significant difference noted between categories. Overall scores did not differ significantly across models, but the contextualized model had a significantly lower mean score in the Treatment domain (19.8 ± 3.4) compared to the input-output model (24.9 ± 1.7, *p* = 0.007) and the chain of thought with instruction-tuning model (25.2 ± 2.2, *p* = 0.004). There were no significant differences in any of the quality domains between the four models.

## 4 Discussion

This is the first study to the authors’ best knowledge, that shows that prompt engineering can be used to generate responses to frequently asked questions in implant dentistry, covering areas related to identifying ideal candidates for implant therapy, therapeutic aspects, recognizing symptoms of peri-implant disease, and implant maintenance. Unlike previous LLM research in dentistry which focused on standardized exam questions (19, 20, 41–44), this study explored realistic scenarios where individuals may use chatbots to seek guidance on dental implant therapy.

Developing LLMs in healthcare has relied on fine-tuning, also known as model adaptation, where the LLM is retrained on specialized datasets to improving its performance. However, this can be computationally intensive as it requires optimizing numerous parameters, which requires significant cost and time (24). Prompt engineering offers an accessible and cost-effective means to customize responses. This study shows that using prompt engineering can achieve mixed performances in answering frequently asked questions by patients, and results may vary depending on the type of questions queried. Compared to other models, the contextualized model performed less effectively for questions in the Treatment domain. This was a surprising outcome given the knowledge base it was augmented with were S3 Level Clinical Practice Guidelines developed under the European Federation of Periodontology, which is intended to support decision-making in patient treatment based on the best available evidence. The discrepancy may be due to the highly patient-specific focus on some questions in this study, while the S3 guidelines were written for clinicians to guide their treatment decisions and advice to patients. This study also found that there were trade-offs in quality domains depending on the model. The contextualized model had the best scores in when providing reliable sources to support its answers, as it relied on the recently developed S3 guidelines. In contrast, the relatively high fail rates of the other models were attributed to frequent issues such as misquoting references or citing them out of context. However, the contextualized model performed worse in the clarity and relevance of its responses. This may be because its attempt to incorporate relevant sources led to overly complex and convoluted answers, reducing overall comprehensibility to non-clinicians. The contextualized model may have struggled to provide nuanced responses that matched patient concerns. By prioritizing incorporating reliable references, this may have come at the cost of clarity and direct relevance to the questions asked, as compared to the other models.

The findings of this study are in agreement with others which found that GPT models may struggle in providing personalized and clear advice to patients (45), and may produce significant errors in highly specialized aspects of clinical care (46, 47). Well-engineered prompts can produce more comprehensive and accurate responses (25, 48, 49). Despite these challenges, this study supports existing evidence that LLMs are valuable tools for dental education, particularly in dental implantology (50–52). However, their reliability and usefulness may vary between models (e.g., Google Gemini vs. GPT-3.5/GPT-4), and may exhibit bias when discussing different implant brands (50, 51).

The language-based structure of LLMs may also mean that when a topic is under-resourced, it may compensate by drawing on semantically similar concepts from related but distinct areas, resulting in potential inaccuracies. This is known as representational heuristic bias, and is a type of cognitive bias, where LLMs generalize information from related concepts (53, 54). LLMs are also prone to other cognitive biases such as false consensus bias, where responses are generated on what the model assumes is the most popular opinion, and frequency bias, where responses are skewed towards more common diagnoses and treatments (55). These biases may be mitigated in implant dentistry due to abundant and specific training data available. However, these findings suggest that generating responses to commonly asked questions by patients in dentistry requires thorough evaluation given the varied levels of resource representation across different specialties. Furthermore, the data on which the LLM was trained on may not fully represent diverse populations. For example, all questions were posed in English, limiting the applicability of these results to non-English speaker, or those with lower health literacy. Patients with lower literacy levels may struggle to understand technical explanations, potentially limiting its accessibility.

This study is not without its limitations. Only four types of prompt engineering were tested. Other types of prompt strategies that could be useful in LLM applications in dentistry include reflection of thoughts prompting, which involves guiding the LLM to break down the task into sequential steps and backtracking prior steps for further reflection (25). Another type is known as tree of thoughts prompting, which aims to explore multiple reasoning paths (56). These were not utilized in this current study as these techniques may be more suited for more complex tasks that require extensive reasoning. Another important limitation is the constantly evolving nature of dental implant literature. Certain promising procedures may not yet be supported by well-conducted randomized controlled trials nor addressed in consensus statements. Additionally, the study utilized 30 questions as part of its exploratory nature. However, future studies should consider incorporating a broader set of questions, including those aligned with the Implant Dentistry Core Outcome Set and Measurement (ID-COSM) domains, to ensure comprehensive assessment (51). Additionally, even though the raters assessed each model three times with a wash-out period, there is still potential for bias as the same raters rated it. Another limitation is that only the GPT-4-o model was used. Comparisons with other LLMs, such as Google Gemini, Claude, and DeepSeek, would provide a more comprehensive analysis of prompt engineering performance. For example, Google Gemini has been noted for its safety features in recommending professional dental care, and its ability to incorporate graphical elements in responses, which may contribute to a better end-user experience (51, 52). Considerations for further research include using prompt engineering to confine responses within a specific timeframe to prevent GPTs from referencing outdated information (i.e., historical bias), or to only include high-quality academic publications as a reference source. Another promising approach is training LLMs on specialized biomedical corpora to enhance its understanding of current and domain-specific practices to improve its accuracy (24). For example, PerioGPT, a fine-tuned version of GPT-4o tailored for periodontal queries, demonstrated significantly improved performance compared to general-purpose models. This suggests that AI-driven implant dentistry education could benefit from similar fine-tuning approaches to enhance accuracy and domain relevance (57). This is crucial as general-purpose GPT models are only trained on publicly available data and may not have access to latest research (58). Further work is required to evaluate these models with patient volunteers in real-world settings before considering its adoption as part of routine clinical care (59). Clinical decisions often involve assessing potential patient benefit, understanding the level of being informed of the patient, clinical expertise, and interpreting limited evidence (60, 61). These require nuanced clinical judgement, which GPTs may not fully replicate.

Furthermore, research is needed to assess the GPT's effectiveness across different literacy levels and language barriers, as health literacy is a stronger predictor of health outcomes than age, income, or education (62). Ethical AI development is crucial in preventing the reinforcement of existing healthcare disparities, ensuring transparency, accountability, and equity, particularly for underrepresented populations (63, 64). Comprehensive data documentation and systematic identification of algorithmic biases are necessary to improve transparency in LLM models and ensure appropriate representation of diverse populations (65). Ethical evaluations should be systematically integrated into model development to mitigate risks, ensure fairness and inclusivity by incorporating stakeholder involvement, and training models on diverse, representative data, including vulnerable groups (65, 66). In this context, GPTs for patient dental education should be designed to be accessible and consider varying levels of health literacy and language proficiency amongst participants. The performance of LLMs in voice interactions, which could be beneficial for individuals with disabilities, such as those with visual impairments or motor limitations that affect typing may also be evaluated.

Clinically, GPTs can enhance efficiency by reducing administrative workload, such as answering patient queries and minimizing the burden on clinicians and administrators making follow-up calls. It can be integrated into clinical workflows before a consultation, or at subsequent visits for further patient clarification. Importantly, GPTs have the potential to reduce the power differential between clinician and patient by providing accessible, high-quality information, thereby strengthening shared decision-making and bridging information gaps (67, 68). However, human oversight remains essential to ensure accuracy, prevent errors and maintain patient trust.

## 5 Conclusion

Prompt engineering is a promising approach in enhancing responses to frequently asked questions in implant dentistry. State-of-the-art GPTs can potentially be used to inform patients about dental implants, reducing the knowledge gap between dentists and patients, and empowering the latter to make more informed decisions. This is valuable because implant dentistry, while offering significant benefits in the rehabilitation of edentulous patients, is a procedure that can carry significant post-surgical risks and long-term complications such as peri-implantitis.

Providing reliable information for patients is important as GPTs may draw from open internet sources. Integrating contextual knowledge to a GPT using an API that integrates high-quality dental information offers a potential solution. However, further work is required to improve the clarity and relevance of answers when a contextualized model is used. Quality of responses varies across different prompt designs. While GPT-based information is not a substitute for clinical advice, these models show potential as supportive tools in patient education.

## Data Availability

The raw data supporting the conclusions of this article will be made available by the authors, without undue reservation.
