# Supplementary material for: Enhancing patient-centered information on implant dentistry through prompt engineering: a comparison of four large language models
Source: Front Oral Health. 2025 Apr 7;6:1566221. doi: 10.3389/froh.2025.1566221 (PMC12009804; doi:10.3389/froh.2025.1566221)
Supplement: Supplementary file 1 [file Table1.docx]

**Supplementary Table 1.** Prompts for the four GPT models.

| **Input-output prompting** |
| --- |
| “I would like you to assume the role of a general dentist. Review the question and explain clearly and succinctly as you would to a patient. Provide references to support the health information presented.” |
| **Zero-shot-chain of thought prompting** |
| “I would like you to assume the role of a general dentist. Review the question and explain clearly and succinctly as you would to a patient. Complete the task above step-by-step. Provide references to support the health information presented.” |
| **Zero-shot chain of thought prompting with instruction-tuning and temperature control** |
| “I would like you to assume the role of a general dentist. Review the question and explain clearly and succinctly as you would to a patient. Provide references to support the health information presented. Do not make up an answer that you do not know.” |
| **Contextualised** |
| “I would like you to assume the role of a general dentist. Please refer to the S3 guidelines attached. Provide references to support the health information presented.” |
