# Supplementary material for: Enhancing patient-centered information on implant dentistry through prompt engineering: a comparison of four large language models
Source: Front Oral Health. 2025 Apr 7;6:1566221. doi: 10.3389/froh.2025.1566221 (PMC12009804; doi:10.3389/froh.2025.1566221)
Supplement: Supplementary file 2 [file Table2.docx]

**Supplementary Table 2.** Comparison of model performance based on question and quality domains (average scores).

|  | Input-output model  (Mean (SD)) | Chain of thought model  (Mean (SD)) | Instruction-tuning model (Mean (SD)) | Contextualized model  (Mean (SD)) | p-value* |
| --- | --- | --- | --- | --- | --- |
| Overall score | 25.22 (2.41) | 25.23 (2.75) | 24.97 (2.55) | 24.35 (4.13) | 0.933 |
| Question Domain |  |  |  |  |  |
| Patient Selection | 25.21 (3.08) | 24.29 (3.59) | 23.43 (4.14) | 25.5 (3.51) | 0.837 |
| Associated Risks | 24.12 (1.11) | 26.12 (1.49) | 25 (0.82) | 24.75 (6.33) | 0.245 |
| Symptoms | 26.83 (1.04) | 27.83 (0.29) | 25.33 (0.76) | 27.67 (2.75) | 0.228 |
| Treatment | 26.35 (1.29) | 24.95 (2.20) | 26.3 (1.55) | 21.5 (3.40) | 0.002** |
| Prevention | 25.00 (2.29) | 25.83 (2.75) | 25.83 (1.89) | 26.5 (2.60) | 0.844 |
| Prognosis | 21.50 (2.60) | 24.00 (4.44) | 22.83 (1.26) | 25.17 (3.75) | 0.538 |
| Quality Domain |  |  |  |  |  |
| Accuracy | 4.48 (0.50) | 4.42 (0.64) | 4.40 (0.55) | 4.43 (0.58) | 0.916 |
| Clarity | 4.77 (0.25) | 4.72 (0.36) | 4.68 (0.36) | 3.92 (0.60) | <0.001** |
| Relevance | 4.58 (0.35) | 4.43 (0.49) | 4.50 (0.44) | 3.95 (1.03) | 0.099 |
| Completeness | 4.15 (0.54) | 4.27 (0.52) | 4.15 (0.49) | 3.97 (0.97) | 0.766 |
| Sources | 2.83 (1.11) | 3.02 (1.16) | 2.90 (1.08) | 4.10 (1.00) | <0.001** |
| Usefulness | 4.40 (0.48) | 4.38 (0.54) | 4.33 (0.55) | 3.98 (0.93) | 0.293 |

*Kruskal-Wallis rank sum test.

**Pairwise significant differences (Dunn’s test, adjusted p-values):

Treatment: Input-output vs Contextualized (p=0.0036), Instruction-tuning vs Contextualized (p=0.0051).

Clarity: Input-output vs Contextualized (p <0.001), Chain of thought vs Contextualized (p<0.001), Instruction-tuning vs Contextualized (p<0.001).

Sources: Input-output vs Contextualized (p <0.001), Chain of thought vs Contextualized (p<0.001), Instruction-tuning vs Contextualized (p<0.001).
