# Supplementary material for: Enhancing patient-centered information on implant dentistry through prompt engineering: a comparison of four large language models
Source: Front Oral Health. 2025 Apr 7;6:1566221. doi: 10.3389/froh.2025.1566221 (PMC12009804; doi:10.3389/froh.2025.1566221)
Supplement: Supplementary file 3 [file Table3.docx]

**Supplementary Table 3.** Comparison of model performance based on question and quality domains (based on pass rates).

|  | Input-output model  (Proportion (95% CI)) | Chain of thought model  (Proportion (95% CI)) | Instruction-tuning model (Proportion (95% CI)) | Contextualized model  (Proportion (95% CI)) | p-value* |
| --- | --- | --- | --- | --- | --- |
| Question Domain |  |  |  |  |  |
| Patient Selection | 0.80  (0.70 - 0.87) | 0.76  (0.66 - 0.84) | 0.76  (0.66 - 0.84) | 0.82  (0.73 - 0.89) | 0.732 |
| Associated Risks | 0.81  (0.68 - 0.90) | 0.92  (0.80 - 0.97) | 0.88  (0.75 - 0.94) | 0.81  (0.68 - 0.90) | 0.386 |
| Symptoms | 0.86  (0.71 - 0.94) | 0.83  (0.68 - 0.92) | 0.81  (0.65 - 0.90) | 1.00  (0.90 - 1.00) | 0.029 |
| Treatment | 0.88  (0.81 - 0.93) | 0.82  (0.74 - 0.88) | 0.89  (0.82 - 0.94) | 0.58  (0.49 - 0.67) | <0.001** |
| Prevention | 0.92  (0.78 - 0.97) | 0.94  (0.82 - 0.98) | 0.89  (0.75 - 0.96) | 0.97  (0.86 - 1.00) | 0.697 |
| Prognosis | 0.67  (0.50 - 0.80) | 0.81  (0.65 - 0.90) | 0.83  (0.68 - 0.92) | 0.86  (0.71 - 0.94) | 0.222 |
| Quality Domain |  |  |  |  |  |
| Accuracy | 0.93  (0.84 - 0.97) | 0.90  (0.80 - 0.95) | 0.95  (0.86 - 0.98) | 0.90  (0.80 - 0.95) | 0.715 |
| Clarity | 1.00  (0.94 - 1.00) | 0.98  (0.91 - 1.00) | 0.98  (0.91 - 1.00) | 0.80  (0.68 - 0.88) | <0.001** |
| Relevance | 1.00  (0.94 - 1.00) | 0.95  (0.86 - 0.98) | 0.97  (0.89 - 0.99) | 0.70  (0.57 - 0.80) | <0.001** |
| Completeness | 0.87  (0.76 - 0.93) | 0.90  (0.80 - 0.95) | 0.93  (0.84 - 0.97) | 0.73  (0.61 - 0.83) | 0.014** |
| Sources | 0.25  (0.16 - 0.37) | 0.32  (0.21 - 0.44) | 0.28  (0.19 - 0.41) | 0.78  (0.66 - 0.87) | <0.001** |
| Usefulness | 0.95  (0.86 - 0.98) | 0.93  (0.84 - 0.97) | 0.95  (0.86 - 0.98) | 0.75  (0.63 - 0.84) | 0.001** |

*Fisher’s exact test.

**Pairwise significant differences (Fisher’s exact test, Bonferroni-adjusted p-values):

Treatment: Input-output vs Contextualized (p <0.001), Chain of thought vs Contextualized (p<0.001), Instruction-tuning vs Contextualized (p<0.001).

Clarity: Input-output vs Contextualized (p=0.002), Chain of thought vs Contextualized (p=0.012), Instruction-tuning vs Contextualized (p=0.012).

Completeness: Instruction-tuning vs Contextualized (p=0.036).

Relevance: Input-output vs Contextualized (p <0.001), Chain of thought vs Contextualized (p=0.003), Instruction-tuning vs Contextualized (p <0.001).

Sources: Input-output vs Contextualized (p <0.001), Chain of thought vs Contextualized (p<0.001), Instruction-tuning vs Contextualized (p<0.001).

Usefulness: Input-output vs Contextualized (p=0.023), Instruction-tuning vs Contextualized (p=0.023).
